# *Coprinopsis cinerea* Galectin CGL1 Induces Apoptosis and Inhibits Tumor Growth in Colorectal Cancer Cells

**DOI:** 10.3390/ijms24010235

**Published:** 2022-12-23

**Authors:** Mengli Yan, Yaxuan Chen, Mengke Li, Jiamin Wu, Zemin Fang, Junjun Wang, Juanjuan Liu

**Affiliations:** 1 School of Life Sciences, Anhui University, Hefei 230601, China; 2 Anhui Key Laboratory of Modern Biomanufacturing, Hefei 230601, China; 3 Anhui Provincial Engineering Technology Research Center of Microorganisms and Biocatalysis, Hefei 230601, China; 4 High Magnetic Field Laboratory, Chinese Academy of Sciences, Hefei 230031, China

**Keywords:** *Coprinopsis cinerea*, galectin, CGL1, colorectal cancer, apoptosis

## Abstract

Mushroom galectins are promising anticancer agents for their low *IC*_50_ values against cancer cells in vitro. In this study, two *Coprinopsis cinerea* galectins, CGL1 and CGL2, were heterologously expressed, and their biochemistry properties and anticancer effects were evaluated. The purified galectins were thermostable at neutral pH conditions. They both existed as tetramers and shared a high affinity towards lactose. CGL1 and CGL2 strongly inhibited the cell viability of many cancer cell lines, including three colorectal cancer cells, in a dose-dependent manner by inducing mitochondria-mediated caspase-dependent apoptosis. Furthermore, CGL1 exhibited higher apoptosis-inducing ability and cytotoxicity than CGL2. In vivo cell viability experiments based on two xenograft mouse models showed that CGL1 had a more substantial inhibitory effect than CGL2 on HCT116 tumor growth (*p* < 0.0001), whereas only CGL1 inhibited DLD1 tumor growth (*p* < 0.01). This is the first study to evaluate the anti-colorectal cancer effect of mushroom lectins in vivo, and our results showed that CGL1 is a potent agent for colorectal cancer treatment.

## 1. Introduction

Lectins are a family of carbohydrate epitopes-binding proteins of non-immune origin with at least one specific and reverse carbohydrate-recognition domain. In nature, lectins distribute widely among plants, animals, viruses, bacteria, fungi, and humans [1], with 82% of fungal lectins from mushrooms [2]. Increasing evidence has proved that mushrooms are rich sources of novel lectins with unique carbohydrate specificities and diverse physiological functions, including serving as storage nutrients [3], taking part in fungal development [4,5], and mediating defense systems [3,6,7].

Due to the demand for novel anticancer drugs, increasing attention has been paid to mushroom lectins as potential medicines in cancer treatment. In vitro anticancer activities upon different types of cell lines are demonstrated in lectins from various mushrooms, including *Pholiota adipose* [8], *Hericium erinaceus* [9], *Lactarius flavidulus* [10], and *Agaricus bisporus* [11]. The anticancer mechanisms of some mushroom lectins were explored in vitro, and they mainly act by inhibiting DNA synthesis or inducing apoptosis in cells. For example, *A*. *bisporus* ABL reduces the S-phase cell population and suppresses human RPE cell proliferation [12]. Further signal transduction pathway analysis shows Erk and Akt hypophosphorylation is related to cell cycle arrest [12]. In addition, AAL from *Agrocybe aegerita* can translocate to the nucleus of human HeLa cells and exhibit cytotoxicity by initiating apoptosis [13,14]. However, the molecular pathway of how apoptosis is induced is still under investigation.

Colorectal cancer is one of the most common malignant neoplasia due to its high prevalence and mortality rate. The low *IC*_50_ values (0.1–1 µM) of mushroom lectins against colorectal HT29 cell lines [15,16] and SW-480 cell lines [17] indicate their potential usage in anti-colorectal adenocarcinoma therapy. Although several in vivo studies have evaluated the anti-hepatocellular carcinoma and anti-sarcoma effect of mushroom lectins, including AAL [14] and *Pleurotus ostreatus* POL [18], and the anti-melanoma effect of *Boletus edulis* BEL *β*-Trefoil [19], no anti-colorectal cancer effect has been reported in vivo for mushroom lectins. Thus, it is of great value to disclose the in vivo biological activity of mushroom lectins on colorectal cancers.

Based on the amino acid sequences and three-dimensional structures, mushroom lectins are classified into cyanovirin-N-like fold lectins, galectins, *β*-trefoil fold lectins, yeast adhesion fold lectins, actinoporin-like fold lectins, and *β*-propeller fold lectins [2]. Among the six groups of lections, galectins are a conserved family of *β*-galactoside-binding proteins. A *β*-sandwich structure formed by two parallel six-stranded antiparallel *β*-sheets, similar to galectins of plant and animal origin, is recognized as the feature of mushroom galectins according to the four reported structures. Several mushroom galectins have been characterized, including *Coprinopsis cinerea* CGL2 and CGL3 [20,21], *Agrocybe cylindracea* ACG [22], and *A*. *aegerita* AAL [23]. They usually form oligomers to be bioactive and function in different oligomerization states [24,25]. For example, AAL is dimeric as other reported galectins, but CGL2 tends to be tetrameric, assembling as four-leafed clover [20,23].

The mushroom *C*. *cinerea* galectins CGL1 and CGL2 share 86% identity [26]. Based on the crystal structures, five amino acid residues in CGL2, including His-51, Arg-55, Asn-64, Trp-72, and Glu-75, are demonstrated to bind to the *β*-galactoside of the sugar substrates [26]. Though the two galectins can be induced to express by nematodes or nutrient limitation [27,28], only CGL2-mediated nematotoxicity is proved to depend on the interaction between CGL2 and a *β*-galactoside on the core of N-glycans in the *Caenorhabditis elegans* intestine [29]. Furthermore, CGL1 and CGL2 are proposed to play different biological functions since they show differential expression patterns during *C*. *cinerea* fruiting body formation [30]. However, the specific biological effect of CGL1 has not been described, and it is unclear how the two galectins function differently.

This study demonstrated that CGL1 and CGL2 shared tetrameric structures, and both galectins were thermostable and exhibited maximum activities under neutral and alkaline conditions. CGL1 and CGL2 had inhibitory effects on many cancer cells in vitro, especially on the colorectal cancer cell lines HCT116 and DLD1, via inducing apoptosis. Furthermore, in vivo studies showed that CGL1 significantly decreased the subcutaneous tumor volume and weight in HCT116- and DLD1-bearing mice after injection for two weeks. By comparison, CGL2 injection slightly inhibited the HCT116-bearing mice tumor growth but with no effect on DLD1-bearing mice. As such, combining the in vitro and in vivo results, CGL1 has remarkable application potential in colorectal cancer therapy.

## 2. Results

### 2.1. CGL1 and CGL2 Expression, Purification, and Biochemical Characterization

**Figure 1:**
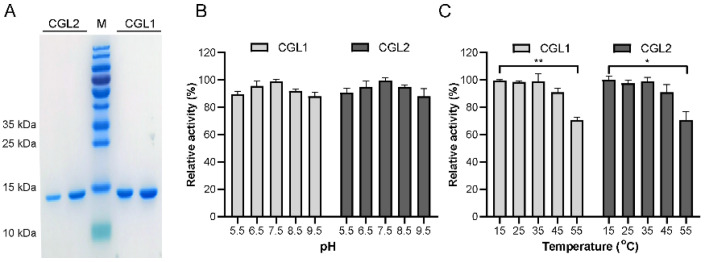
Purification of CGL1 and CGL2 and effects of pHs and temperatures on their stability. (**A**), Coomassie brilliant blue R250 stained 15% SDS-PAGE gel showed molecular weights of about 16 kDa for CGL1 and CGL2. (**B**), pH stability at pH 5.5–9.5. Samples were incubated at 4 °C for 24 h. (**C**), Thermostability at 15–55 °C. Samples were incubated at pH 7.0. The data were analyzed using a student’s *t*-test (* *p* < 0.05, ** *p* < 0.01). Data show mean ± SD, *n* = 3.

The full-length *cgl1* and *cgl2* were amplified successfully using the genomic DNA of *C*. *cinerea* as the template since there was no predicted intron in the two genes. The two galectins were heterologously expressed in *E*. *coli* and purified successfully to homogeneity using Ni^2+^-NTA affinity chromatography. Only one band with an apparent molecular weight of about 16 kDa was determined by SDS-PAGE for two galectins, respectively (Figure 1A), in agreement with the calculated molecular weight and that of galectins purified by lactose-affinity chromatography from *C*. *cinerea* [31]. Furthermore, CGL1 and CGL2 exhibited typical haemagglutination activity on rabbit erythrocytes at the lowest concentration of 1.0 µg/mL after incubation for 1 h.

CGL1 and CGL2 displayed the highest activity at pH 6.5–8.5 and remained above 80% of activity between pH 6.0 and 9.5 (Figure S1A). The optimal temperature of CGL1 and CGL2 was 25 °C (Figure S1B). CGL1 and CGL2 were highly stable at pHs between 5.5–8.5, with 90% of the original activities remaining after 24 h (Figure 1B). They were stable at temperatures lower than 55 °C and lost about 30% of their original activities after 24 h incubation at 55 °C (Figure 1C).

### 2.2. CGL1 and CGL2 Share a Similar Tetrameric State and Lactose Binding Affinity

**Figure 2:**
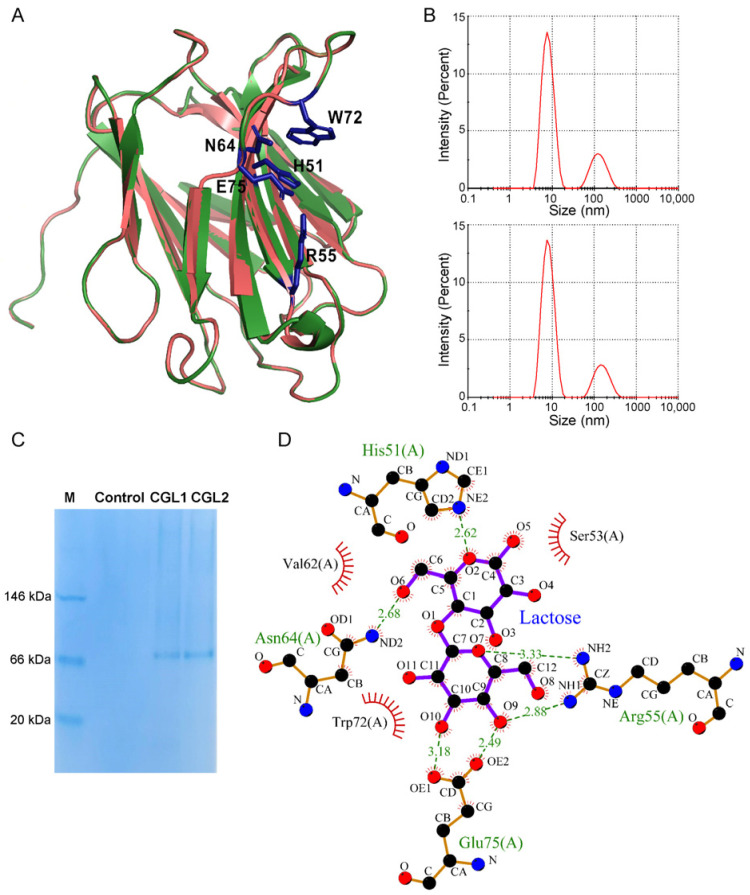
Structure modeling and oligomerization state identification of CGL1. (**A**), Computational modeling of CGL1 structure using CGL2 as the template. CGL1 and CGL2 were displayed in green and red, respectively. (**B**), Dynamic light scattering analysis of CGL1 and CGL2. (**C**), Native-PAGE of CGL1 and CGL2, using the purified pET22b (+) vector protein as the control. (**D**), Computational modeling of CGL1 interacted with the substrate lactose.

The homology modeling data using CGL2 [20] as the template suggested that the three-dimensional structure of CGL1 was represented as a tetrameric state like CGL2 (Figure 2A and Figure S2). From the dynamic light scattering (DLS) analysis, CGL1 and CGL2 showed a predominant peak at a diameter of about 7.53 nm (Figure 2B). The calculated molecular weight of proteins at the predominant peaks was about 64 kDa, suggesting their tetrameric states. These results were also consistent with the Native-PAGE analysis, in which CGL1 and CGL2 also existed as tetramers (Figure 2C). Furthermore, as shown in Figure 2A, some residues in *β* strands of CGL1 were different from CGL2, including I11, A21, T24, A60, V78, L94, L103, T113, S126, and I140. Other different residues were in loops (D15, A19, V29, T67, G69, G82, K85, G86, N106, D107, and S109).

CGL1 and CGL2 shared similar lactose binding residues and hydrogen bond networks based on the bioinformatic analysis (Figure 2A,D; [20]). His-51, Arg-55, Asn-64, Trp-72, and Glu-75 are five sugar-binding residues in CGL1. Furthermore, a quartet of salt-bridged arginine and glutamate residues formed the edge of the binding site and clustered as a platform of hydrogen bonding partners to ligand hydroxyl groups at the reducing end of lactose (Figure 2D; [20]). Some residues that differed between CGL1 and CGL2 were located on the same *β* strands as sugar-binding residues. Some were located on the connecting loops, creating a binding cleft open to both sides. Isothermal calorimetric titration (ITC) analysis demonstrated a similar binding affinity of lactose to CGL1 and CGL2. The binding constants (*K*) of the complexes for CGL1 and CGL2 were 2.74 × 10^−4^ M and 2.83 × 10^−4^ M. The change in free energy of binding (Δ*G*) was −6.06 kcal/mol and −6.07 kcal/mol, respectively. As such, the different residues did not lead to the different oligomerization states and lactose binding abilities between CGL1 and CGL2.

### 2.3. CGL1 Shows a Better Inhibitory Effect on Cancer Cells than CGL2 In Vitro

**Figure 3:**
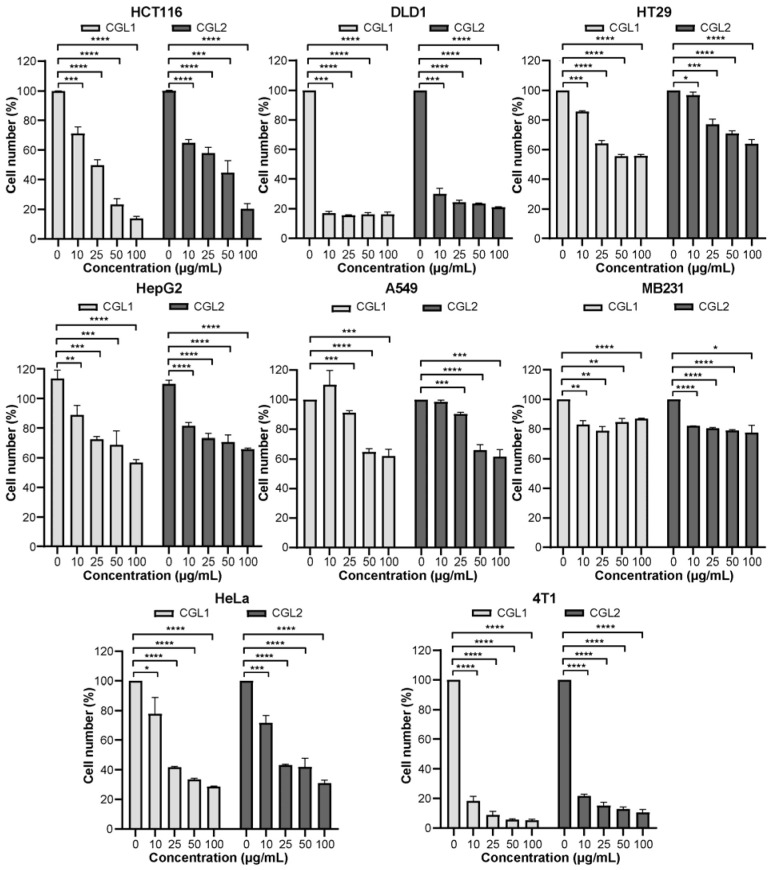
CGL1 shows a better inhibitory effect on cancer cells than CGL2 in vitro. Cancer cells HCT116, DLD1, HT29, HepG2, A549, MB231, HeLa, and 4T1 were incubated with serials concentrations of CGL1 or CGL2 for 16 h, and then the cell viabilities were detected. The data were analyzed using a student’s *t*-test (* *p* < 0.05, ** *p* < 0.01, *** *p* < 0.001, **** *p* < 0.0001). Data show mean ± SD, *n* = 3.

CGL1 and CGL2 exhibited toxicity on cancer cells HCT116, DLD1, HT29, HepG2, A549, MB231, HeLa, and 4T1 in vitro in a dose-dependent manner (Figure 3). CGL1 was more toxic than CGL2. Aberrant glycosylation constitutes a hallmark of epithelial tumor progression, and N-glycans and O-glycans are considered targets for cancer therapy [32,33]. Among the eight cancer cells, the two galectins exhibited weaker cytotoxicity on HepG2, A549, MB231, and HeLa cell lines. This might be due to the largest proportion of core-fucosylated glycans in HeLa cells, especially in HepG2 cells [34,35], and reduced terminal galactose residues but increased sialylated glycans in A549 cells [36] and increased heparin sulfate proteoglycans in MB231 cells [37]. For colorectal cancer cells, LacdiNAc (GalNAc*β*1, 4GlcNAc*β*1-) has been shown to play critical roles during tumor progression but depends on the differentiation and mutational status [33,38]. Compared with differentiated and highly proliferated HT29 with *RAS* and *p53* mutations, HCT116 is undifferentiated with a *RAS* mutation and shares a higher level of LacdiNAc [33]. Relatively, glycoproteins of DLD1 were less reported. Its highly sensitive to sulfated and acetylated heteropolysaccharides containing fucose, galactose, mannose, and glucose than HCT116 and HT29, suggesting it might be of complex glycosylation [39,40]. Particularly, all three colorectal cancer cells were sensitive to the two galectins here. For example, the cell viability of DLD1 was less than 20% when treated with 10 μg/mL galectins (*IC*_50_ < 1 μM). The *IC*_50_ values of CGL1 and CGL2 on HCT116 cells were 22.13 μg/mL (1.35 μM) and 29.09 μg/mL (1.77 μM), respectively. On the other hand, CGL1 and CGL2 also showed cytotoxicity on two normal cells, HL7702 and NIH-3T3, but with higher *IC*_50_ values than DLD1 and HCT116 (Figure S3). Further PI/Calcein-AM staining demonstrated that after 16 h incubation with 100 μg/mL CGL1 or CGL2, nearly all HCT116 cells died (Figure S4). These results suggested that CGL1 and CGL2 can induce human cancer cell death, including different types of human colorectal cancer cells.

### 2.4. CGL1 and CGL2 Induce Human Colorectal Cancer Cells Apoptosis

**Figure 4:**
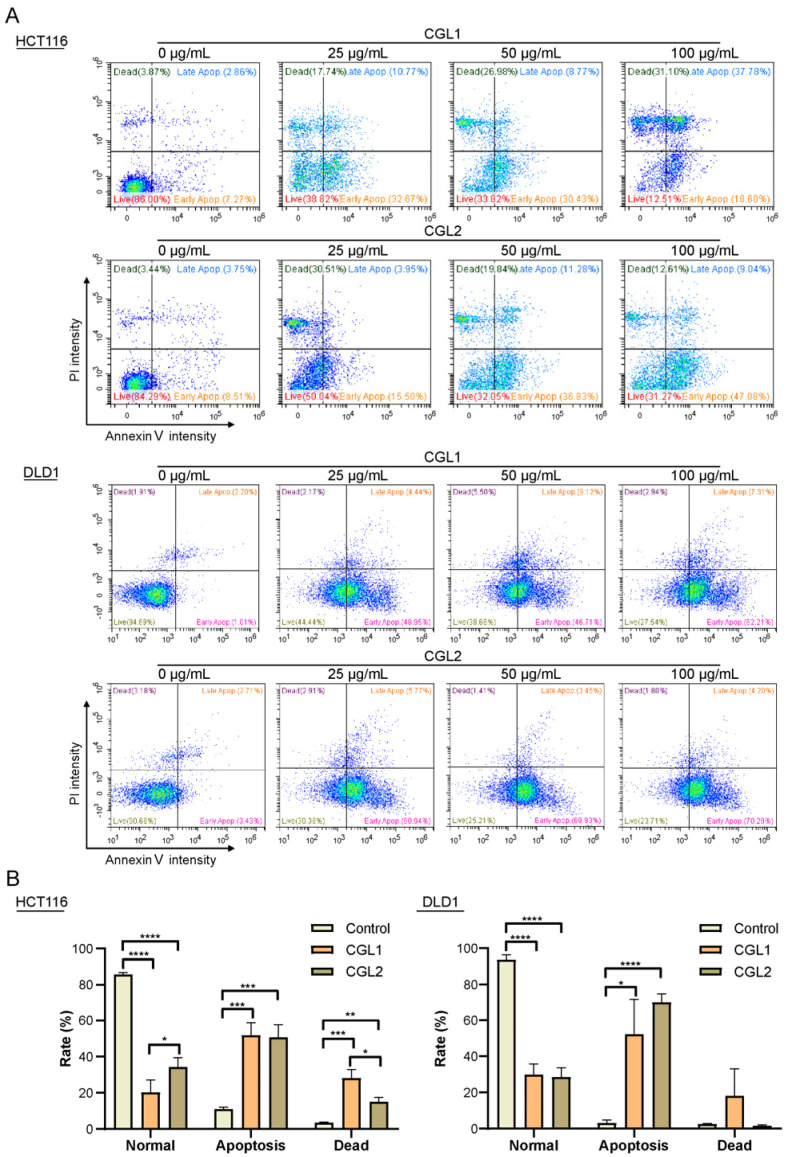
CGL1 and CGL2 induce HCT116 and DLD1 cells apoptosis. HCT116 ((**A**), the **upper panel**) and DLD1 ((**A**), the **lower panel**) cells were incubated with serials concentrations of CGL1 and CGL2 for 16 h, dually labeled with Annexin and PI, and then analyzed using flow cytometry. The ratios of normal, apoptosis, and dead HCT116 ((**B**), the **left panel**) and DLD1 ((**B**), the **right panel**) cells after being treated with 100 µg/mL CGL1 or CGL2 for 16 h were analyzed using a student’s *t*-test (* *p* < 0.05, ** *p* < 0.01, *** *p* < 0.001, **** *p* < 0.0001). Data show mean ± SD, *n* = 3.

Some apoptotic morphological phenotypes, including cell volume reduction and rounding, were observed after incubating the cancer cells with CGL1 or CGL2. Thus, two colorectal cancer cells, HCT116 and DLD1, were further chosen to identify apoptotic ratios using Annexin V and PI dual-labeling and flow cytometry. After incubation with 25, 50, and 100 μg/mL CGL1 or CGL2, the apoptotic and dead cells increased in a dose-dependent manner (Figure 4). At a concentration of 100 μg/mL CGL1 and CGL2, the apoptotic/dead HCT116 cell percentages were 79.8 ± 6.83% and 65.7 ± 4.96% (*p* < 0.05, Figure 4A, the upper panel, and Figure 4B, the left panel), and apoptotic/dead DLD1 cell percentages were 70.2 ± 5.88% and 71.6 ± 5.24%, respectively (Figure 4A, the lower panel, and Figure 4B, the right panel). A significant increase in early and late apoptotic/dead HT29 cells was also observed after CGL1 and CGL2 treatment (Figure S5). Thus, CGL1 and CGL2 can induce apoptosis to cause cell death and inhibit colorectal cancer growth.

### 2.5. CGL1 and CGL2 Induce Apoptosis in a Mitochondria-Mediated Caspase-Dependent Manner

**Figure 5:**
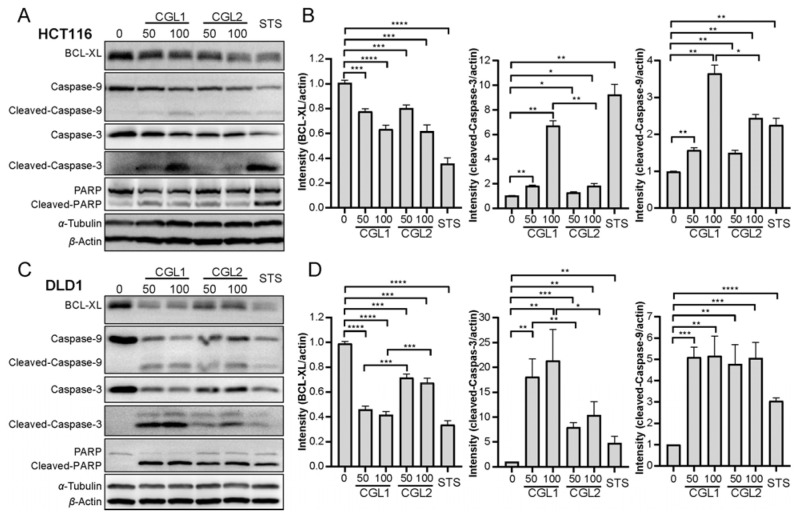
CGL1 and CGL2 induce apoptosis in a mitochondria-mediated, caspase-dependent pathway. (**A**), Western blot analysis of mitochondria-mediated pathways in HCT116 cells treated with 50 µg/mL and 100 µg/mL CGL1 or CGL2 for 16 h. (**B**), Quantification of BCL-XL, cleaved-Caspase-3, and cleaved-Caspase-9 in (**A**). (**C**), Western blot analysis of mitochondria-mediated pathways in DLD1 cells treated with 50 µg/mL and 100 µg/mL CGL1 or CGL2 for 16 h. (**D**), Quantification of BCL-XL, cleaved-Caspase-3, and cleaved-Caspase-9 in (**C**). 500 nM staurosporine (STS) treated cells were used as positive controls. The data were analyzed using a student’s *t*-test (* *p* < 0.05, ** *p* < 0.01, *** *p* < 0.001, **** *p* < 0.0001). Data show mean ± SD, *n* = 3.

The classic apoptotic markers were detected by western blotting to unveil the mechanism by which CGL1 and CGL2 induced cell apoptosis. As shown in Figure 5, the expression of anti-apoptotic protein BCL-XL in HCT116 and DLD1 cells decreased after CGL1 and CGL2 treatment, with CGL1 exhibiting a more significant reduction than CGL2 in DLD1 cells (*p* < 0.001). Correspondingly, the downstream proteins Caspase-3, Caspase-9, and PARP were cleaved. Compared with 100 μg/mL CGL2, the intensities of cleaved-Caspase-3 (*p* < 0.01) and cleaved-Caspase-9 (*p* < 0.05) after 100 μg/mL CGL1 treatment were 3.6-times and 1.5-times higher (Figure 5A,B) in HCT116 cells. Similarly, the cleaved-Caspase-3 intensities of CGL1 were also higher in DLD1 cells at the same concentrations as CGL2 (*p* < 0.001, Figure 5C,D). These results indicated that CGL1 and CGL2 induced apoptosis through a mitochondria-mediated, caspase-dependent pathway.

### 2.6. CGL1 Exhibits Significant Anti-Colorectal Cancer Effects In Vivo

**Figure 6:**
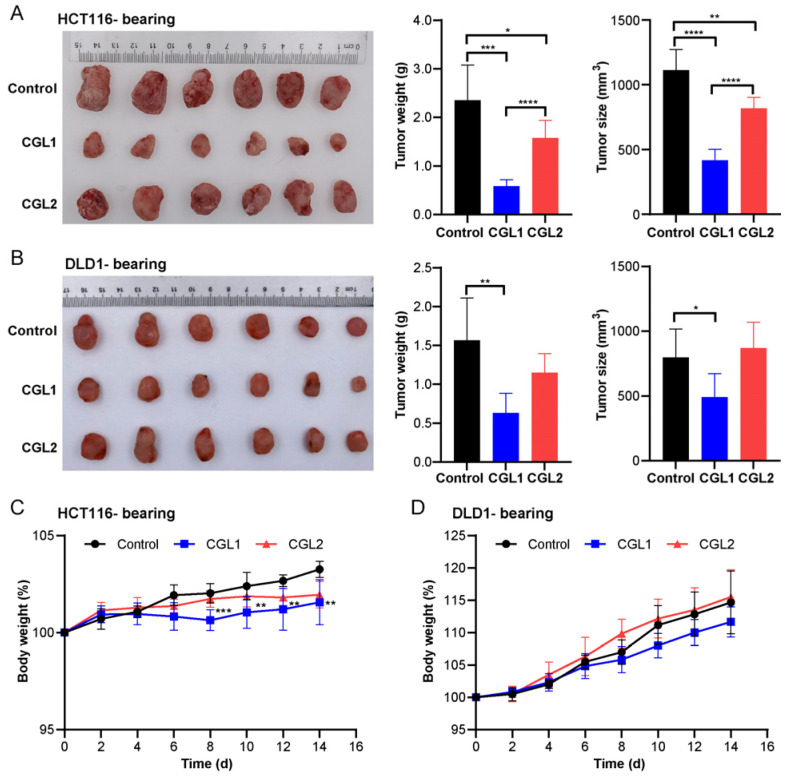
CGL1 exhibits significant anti-colorectal cancer effects in vivo. (**A**,**B**), Typical tumor photographs, tumor weights, and tumor sizes from HCT116-bearing (**A**) or DLD1-bearing (**B**) mice treated with CGL1 and CGL2 on day 14. (**C**,**D**), Curves of average body weights from HCT116-bearing (**C**) or DLD1-bearing (**D**) mice during CGL1 and CGL2 injection. The data were analyzed using a student’s *t*-test (* *p* < 0.05, ** *p* < 0.01, *** *p* < 0.001, **** *p* < 0.001). Control stands for HCT116-bearing or DLD1-bearing mice injected with PBS. Data show mean ± SD, *n* = 6.

Two colon xenograft tumor models were constructed to investigate the in vivo anti-colorectal cancer effects of CGL1 and CGL2. CGL1 inhibited HCT116- and DLD1 tumor xenograft growth, and the tumor size and weight decreased by 62 and 75% in HCT116 and 38 and 59% in DLD1 on day 14, respectively (Figure 6A,B). CGL2 treatment only had an inhibitory effect on HCT116 tumor xenograft growth, with a 26% decrease in tumor size and a 33% decrease in tumor weight, but not on DLD1 tumor xenograft growth. There was no significant difference in food and water consumption between the three groups in each model. Perhaps due to the loss of tumor weight, the CGL1 treatment group showed a significant decrease in body weight from the 8th day compared with the control of HCT116-bearing models (Figure 6C, *p* < 0.001 or *p* < 0.01). However, the body weights in other groups showed no change (Figure 6C,D). Furthermore, both CGL1 and CGL2 injections did not impact mice tissues such as livers, kidneys, and spleens (Figure S6).

## 3. Discussion

Mushroom lectins are potential candidates for anticancer drugs. They mostly exhibit an *IC*_50_ value range of 2–20 µM on cancer cells in vitro [1]. Notably, the *IC*_50_ values of several reported mushroom lectins on colorectal cancer cells were lower than 2 µM [16,17,41]. For example, galectin AAL has been demonstrated to have anticancer activities against seven cancer cell lines, such as human colon adenocarcinoma cell line SW480, human cervical carcinoma cell line HeLa, and mouse sarcoma-180 cell line, and SW480 is the most sensitive to AAL [13,17]. In this study, we heterologously expressed two known galectins, CGL1 and CGL2, from the mushroom *C*. *cinerea*. They exhibited the most potent cytotoxic activity on colorectal cancer cell lines DLD1 (*IC*_50_ values lower than 1 µM) and HCT116 (*IC*_50_ values of 1.35 µM and 1.77 µM, respectively). Thus mushroom lectins, especially galectins, might have better application potential in anti-colorectal adenocarcinoma therapy.

Compared with the in vitro investigations, very few studies reported the in vivo anticancer effect of mushroom lectins. Only several mushroom lectins exhibited more than 70% inhibition ratio on sarcoma-180 or hepatoma H-22 bearing mice, including a novel lectin made up of two homologous *β*-jellyroll domains named POL [18,42], a maltose-binding lectin from *Pleurotus citrinopileatus* [43], and TML-2 from *Tricholoma mongolicum* [44]. AAL is the only mushroom galectin evaluated in vivo using a sarcoma-180-bearing mice model. The inhibition ratio of AAL was 36.36% at a dosage of 0.1 mg/mouse (about 5 mg/kg) upon in situ tumor injection every other day for 20 days [17]. This ratio is similar to CGL2 in our study at a 1.0 mg/kg dosage and treated for 14 days in HCT116 tumor-bearing mice. In contrast, CGL2 treatment for 14 days did not affect DLD1 tumor xenograft growth. Remarkably, CGL1 exhibited a much stronger inhibition effect than AAL and CGL2, with 75 or 60% decreased ratios of tumor weight in two models, indicating that CGL1 has application potential in colorectal cancer treatment. It is important to note that, though both CGL1 and CGL2 showed most sensitive to DLD1 through the three types of colorectal cancer cells in vitro, their anti-cancer effect on HCT116 tumors were actually more obvious than on DLD1 tumors in vivo, like many agents having different anti-cancer effects between in vitro and in vivo.

Studies showed that the biological functions of mushroom galectins are related to their carbohydrate-binding specificities. The anti-predator and anti-parasite effects of CGL2 [29] and CNL [45] are based on their carbohydrate-binding sites that interact with glycan cores on the surface of nematodes and insects. Moreover, the antiproliferative activity of PAL [16] and AAL-2 [14] also depends on specific recognition between carbohydrate-binding sites and carbohydrates on the cell surface. AAL uses carbohydrate-binding sites to bind to the cell membrane, internalize into the cytoplasm, translocate to the nucleus, and induce apoptosis [13]. By comparison, although their carbohydrate-binding sites were identical and their lactose-binding abilities were similar, CGL1 and CGL2 showed different toxicity to HCT116 and DLD1 cells in vitro and in vivo. Similar to our results, the AAL hydrophobic loop region mutant L33A shared the same lactose-binding ability as AAL but lost about half of the ability to enter the nucleus and induce apoptosis. This fact might partly explain our results [46]. The topological integrity of the hydrophobic cluster is required for the full function of AAL, including its apoptosis-inducing ability [46]. Based on the structural modeling and sequence alignment, the 29th residue is the only amino acid that is different between CGL1 and CGL2 in the same hydrophobic ring corresponding to Leu33 of AAL. Compared with the side chains of Val29 in CGL1 and Leu33 in AAL, the side chains of Ala29 in CGL2 and Ala33 in mutant AAL were much smaller, which might affect their interaction with substrates. However, whether this amino acid decisively influences the anti-colorectal cancer effect between CGL1 and CGL2 remains to be further unraveled.

The oligomerization state is another crucial factor affecting the function of mushroom galectin. A mutant I25G at the dimer interface leads to the dissociation of the AAL dimer and loss of apoptosis-inducing activity [46]. The crystal structure of AAL complexed with Thomsen–Friedenreich (TF), a cancer cell proliferation regulator, provides insight into the interaction of a dimerized AAL with TF through a conservative structural motif-based hydrogen bond network [23]. A similar phenotype exists in most mushroom lectins. For example, amino acid mutation at the dimer interface of CCL2 affects its dimerization and reduces its toxicity versus *Caenorhabditis elegans* and *Drosophila melanogaster* [25]. Unlike known mushroom galectins, CGL2 assembles as a tetrameric during function [20,29]. Based on our results, the 29th amino acid difference between CGL1 and CGL2 might not affect tetrameric structure formation in CGL1. Furthermore, the lactose-binding residues, structural motif-based hydrogen bond networks, and lactose-binding constants were similar between CGL1 and CGL2. Thus, their difference in apoptosis induction activity is not relative to the oligomerization states between the two galectins.

## 4. Materials and Methods

### 4.1. Strains and Cultivation Conditions

*C. cinerea* Okayama 7 (#130; *A43*, *B43*, *ade8*) (ATCC No. MYA-4618™) was maintained on YMG agar (yeast malt glucose; per liter, 4 g yeast extract, 10 g malt extract, 4 g glucose, and 15 g agar) plates at 37 °C according to [47]. *Escherichia coli* strains DH5*α* and BL21 (DE3) were cultivated in LB medium (per liter, 10 g tryptone, 5 g yeast extract, 10 g NaCl).

### 4.2. Cell Culture

Human colon cancer cell lines HCT116 and HT29, human liver cancer cell line HepG2, human cervical carcinoma cell line HeLa, human breast adenocarcinoma cell line MB231, and mouse embryonic fibroblast cell NIH-3T3 were cultured in DMEM medium (Corning Life Sciences, Corning, NY, USA), supplemented with 10% (*v*/*v*) fetal bovine serum (Clarkbio, Richmond, VA, USA) and 1% (*v*/*v*) penicillin/streptomycin (Hyclone, Logan, UT, USA). Human colon cancer cell line DLD1, mouse breast adenocarcinoma cell line 4T1, and human normal liver cell HL7702 were cultured in RPMI 1640 medium (Corning Life Sciences, Corning, NY, USA). Human lung adenocarcinoma cell line A549 was cultured in an F12K medium (Corning Life Sciences, Corning, NY, USA). All cells were maintained at 37 °C with 5% CO_2_ in a humidified atmosphere.

### 4.3. Cloning, Heterologous Expression, and Purification of CGL1 and CGL2

The genomic DNA of *C*. *cinerea* was extracted using the Magen Hipure Soil DNA kit (Magen, Guangzhou, China) based on the manufactural instruction and used for gene cloning. The *cgl1* and *cgl2* fragments were amplified using specific primer sets of 1F: CATATGCACCACCACCACCACCACCTCTACCACCTCTTCGTCAACAACCA, 1R: CTCGAGCTAAGCGGGGGGAAGAGGGGGGA; 2F: CATATGCACCACCA CCACCACCACCTCTACCACCTTTTCGTCAACAACCAAG, 2R: CTCGAGCTA AGCAGGGGGAAGTGGGGGGAG, and cloned into pET22b (+) vector (Novagen, Madison, VI, USA), respectively. A His_6_-tag was attached to the N-end of the two proteins to facilitate protein purification. Protein was induced to expression in *E*. *coli* BL21 (DE3) by adding 0.5 mM Isopropyl *β*-D-1-thiogalactopyranoside (IPTG) in LB medium containing 100 mg/L ampicillin when the cell density reached the *OD*_600_ = 2.0. Then the cultures were further incubated at 120 rpm and 16 °C for 16 h. Cells were collected by centrifugation at 5000× *g* for 10 min at 4 °C, resuspended in cold Tris-HCl buffer (30 mM, pH 7.3) containing 150 mM NaCl and 10 mM imidazole, and disrupted by sonication. The supernatant was withdrawn by centrifugation at 12,000× *g* for 30 min and applied to Ni^2+^-NTA affinity chromatography (Novagen, Darmstadt, Germany) to purify CGL1 and CGL2.

The purity of the purified protein was estimated by sodium dodecyl sulfate-polyacrylamide gel electrophoresis (SDS-PAGE) and stained with Coomassie blue R250 (Sangon Biotech, Shanghai, China). The molecular weights of the two galectins were determined by native polyacrylamide gel electrophoresis (Native-PAGE) and actively stained with Coomassie blue R250, using the purified pET22b (+) vector protein as the control. The protein concentration was assayed using the Bradford method with bovine serum albumin as the standard (Sangon Biotech, Shanghai, China).

### 4.4. Haemagglutination Activity (HA) Assay

The HA assay was performed in 96-well microplates. Galectin solutions (50 µL) were serially two-fold diluted in PBS buffer (pH 7.0) before the addition of 50 µL 2% suspension of rabbit erythrocytes (2 × 10^5^ cells, Solarbio, Beijing, China). The HA effects were observed after 1 h incubation at 25 °C using a ZEISS AXIO Scope A1 microscope (ZEISS, Oberkochen, Germany). 1% BSA instead of galectin was used as the control. HA was defined as the reciprocal of the highest dilution exhibiting haemagglutination, reckoning for one haemagglutination unit [48].

### 4.5. Effect of pH and Temperature on HA

The effect of pH on HA was determined in 30 mM citrate-phosphate buffer (pH 5.5–8.5) and 30 mM Tris-HCl (pH 8.5–9.5) at 25 °C. The effect of temperature on HA was determined at pH 7.0 and temperatures ranging from 15–55 °C. The samples were neutralized to pH 7.0 before the HA assay. Then the relative activity at each pH or temperature was calculated using the highest activity in either test as the 100% activity.

pH stability was determined by incubating the samples in different pH buffers at 4 °C for 24 h. Stabilities against temperature were determined by incubating proteins at various temperatures and pH 7.0 for 24 h. The samples were neutralized to pH 7.0 before the residual activities were measured by the HA assay. All experiments were performed in triplicate.

### 4.6. Isothermal Calorimetric Titration (ITC)

ITC was performed using a VP-ITC calorimeter (Microcal Inc., Northampton, MA, USA). CGL1 or CGL2, 50 µM in 1.5 mL PBS buffer (pH 7.0), was titrated with 2–3 µL lactose solution (2 mM in PBS buffer) at an interval of 3 min and a rotation speed of 1000 rpm. The data were fitted via the nonlinear least-squares minimization method to calculate the binding stoichiometry (*N*), binding constant (*K*), change in enthalpy of binding (Δ*H*), and change in entropy (Δ*S*) using Origin software (version 8). The change in free energy of binding (Δ*G*) was calculated using the equation Δ*G* = Δ*H −* T × Δ*S*, where T is the temperature in kelvin [49].

### 4.7. Dynamic Light Scattering (DLS) Analysis

Dilutions of purified CGL1 and CGL2 were filtered through 0.22 µm membranes (Sangon Biotech, Shanghai, China) and detected on a Zetasizer Nano-S device (Malvern, UK) to assay their natively polymerized conformations. *M*, the molecular weight of natively polymerized protein, was estimated using the equation log*R_S_* = −(0.204 ± 0.023) + (0.357 ± 0.005)·log*M*, where *R_S_* is the hydrodynamic radii (the Stokes radius Å) [50].

### 4.8. Cell Viability Assay

HCT116, DLD1, HT29, HepG2, MB231, HeLa, A549, 4T1, HL7702 and NIH-3T3 cells were cultured in 96-well plates at 1 × 10^6^ cells/cm^2^ for 12 h. Then cells were treated with 0, 10, 25, 50, and 100 μg/mL CGL1 or CGL2 for 16 h, respectively. Cell viability was tested using the Cell Titer-Glo luminescent assay according to the Manufactural instructions (Promega, Madison, VI, USA). An envision multilabel plate reader (PerkinElmer, Waltham, MA, USA) was applied to analyze the luminescence intensity.

### 4.9. Annexin V/PI (Propidine Iodide) Apoptosis Detection Assay

HCT116, DLD1, and HT29 cells were plated in 6-well plates at 1 × 10^6^ cells/cm^2^. After 12 h, cells were added with 0, 25, 50, and 100 μg/mL CGL1 or CGL2, respectively, or treated with 500 nM staurosporine for 16 h. All cells were collected 48 h later and washed twice with ice-cold PBS (10 mM, pH 7.2). FITC-Annexin V apoptosis detection kit (KeyGen, Beijing, China) was employed for the assay. Cells were resuspended in 1× binding buffer (10 mM HEPES, 140 mM NaCl, 2.5 mM CaCl_2_, pH 7.4) at a final concentration of 1 × 10^6^ cells/mL before being transferred to 5 mL culture tubes. Next, FITC-Annexin V (5 μL) and PI (5 μL) were added to 100 μL cell solution, mixed, and incubated in the dark for 15 min at room temperature. Then, 400 μL of 1× binding buffer was added before they were analyzed by flow cytometry within 1 h. Data were analyzed using CytExpert (version 2.4.0.28, Beckman, Indianapolis, IN, USA). All experiments were repeated at least 3 times, and representative data were shown in the Figure 4 and Figure S5.

### 4.10. Western Blotting

HCT116 and DLD1 cells plated on 12-well plates were treated with 0, 50, and 100 μg/mL CGL1 or CGL2, or 500 nM staurosporine for 16 h, respectively. Then the cells were washed with PBS and lysed on ice with the M-PER lysis buffer (ThermoFisher, Waltham, MA, USA) supplemented with a protease and phosphatase inhibitor cocktail (Sigma, Saint Louis, MO, USA) at 4 °C. The whole-cell lysate was thermally denatured at 95 °C in 2× SDS loading buffer (20 mM Tris-HCl pH 8.0, 100 mM DTT, 2% SDS, 20% glycerol, and 0.016% bromophenol blue) thoroughly and electrophoresed on 12% SDS-PAGE gels, which were then transferred onto the PVDF membranes by Thermo Scientific Owl VEP-2 (ThermoFisher, Marietta, OH, USA). The membranes were blocked using 5% defatted milk and incubated with corresponding primary antibodies, including Bcl-xl, Caspase-9, Cleaved-Caspase-9, Caspase-3, Cleaved-Caspase-3, *α*-Tubulin, and *β*-Actin, respectively, followed by incubation with the HRP-linked secondary antibodies (1:5000). The visualization of the results was performed using enhanced chemiluminescence Kits (Millipore, MA, USA), and the blots were analyzed using Tanon Fine-do X6 (Tanon, China). Protein levels were normalized to the matching densitometric value of the internal control *β*-Actin by ImageJ software (version 1.42, NIH, Bethesda, MD, USA).

### 4.11. Colorectal Xenograft Tumor Model Study

Four-week-old balb/c female nude mice were purchased from Nanjing Biomedical Research Institute of Nanjing University (Nanjing, China). The mice were acclimatized for one week in a temperature- and humidity-controlled facility, with free access to water and food. All animal welfare and experimental procedures were performed strictly according to the National Institutes of Health Guide for the Care and Use of Laboratory Animals (NIH Publications No. 8023, revised 1978). Additionally, the protocol involving animals was approved by the ethical and humane committee of Anhui University (protocol code 2021-012) and carried out strictly following the related regulations (Hefei, China).

To obtain the subcutaneous xenografts of human colorectal tumors in the mice, HCT116 and DLD1 cells were harvested during exponential growth. Two million HCT116 or DLD1 cells in PBS were suspended in a 1:1 (*v*/*v*) mixture with Matrigel (BD biosciences, East Rutherford, NJ, USA) and injected into the subcutaneous space at the right flank of mice, respectively. When colon tumors reached about 200 mm^3^, mice were randomly divided into three groups, with 6 mice in each group. The purified CGL1 or CGL2 was in situ tumor injected at a 1.0 mg/kg dosage every two days. The control group was treated with 0.9% NaCl. At the same time, the body weight of nude mice was measured daily after galectin treatment during the following 2 weeks. The tumors were weighed, and the tumor volumes were calculated as follows: tumor volume (mm^3^) = [(W^2^ × L)/2], in which width (W) is defined as the smaller of the two measurements and length (L) is defined as the larger of the two measurements [51].

### 4.12. Computational Modeling of CGL1 Structure and Its Interactions with Substrates

The three-dimensional structure of CGL1 was modeled with the Swiss Model (https://swissmodel.expasy.org (accessed on 14 October 2021) using *C*. *cinerea* CGL2 (PDB code: 1UL9) as the template. The structure was further visualized using the Pymol software (version 2.4.1, Delano Scientific LLC, San Carlos, CA, USA). The interaction between CGL1 and the substrate lactose was analyzed by Autodock 4.2 [52]. Firstly, the simulated structure CGL1 was added polar hydrogen atoms, part of the Gasteiger’s charge was added to the lactose atoms, and the rotatable bond was specified. A grid map with 40 × 40 × 40 Å grid points and 0.375 Å grid spacing was generated by Autogrid 4.2 (Autodock software, version 4.2.6, Scripps Research Institute, La Jolla, CA, USA). In the Autodock 4.2 parameter, the number of times the Genetic Algorithm runs was 200, and other docking parameters were set as default values. The best docking poses with the lowest binding energy were shown in Ligplot + v.2.2.4 [53,54], and the interaction analysis of active sites was presented in two dimensions.

### 4.13. Statistical Analyses

All experimental data were presented as mean ± standard deviation (SD). Statistical significance was evaluated by one-way ANOVA followed by Student’s *t*-test with GraphPad Prism 8.2.1 (GraphPad Software, San Diego, CA, USA). *p <* 0.05 was considered statistically significant.

## 5. Conclusions

In conclusion, CGL1 and CGL2 are thermally stable and remain at maximum activities at neutral and alkaline conditions. CGL1 is a tetramer similar to CGL2. They share identical binding residues, motif-based hydrogen bond networks, and binding constants when interacting with lactose. Their application potential in colorectal cancers is explored in vitro by cell cytotoxicity and apoptosis assays and in vivo using the subcutaneous colorectal tumor models. Our results suggest that CGL1 exhibits more substantial effects in colorectal cancer inhibition than CGL2 and is a promising agent for colorectal cancer treatment.

## Data Availability

The datasets used and/or analyzed during the current study are available from the corresponding author upon reasonable request.

## Supplementary Materials

The following supporting information can be downloaded at: https://www.mdpi.com/article/10.3390/ijms24010235/s1.
