# Supplementary material for: Coprinopsis cinerea Galectin CGL1 Induces Apoptosis and Inhibits Tumor Growth in Colorectal Cancer Cells
Source: Int J Mol Sci. 2022 Dec 23;24(1):235. doi: 10.3390/ijms24010235 (PMC9820451; doi:10.3390/ijms24010235)
Supplement: Supplementary file 1 [file ijms-24-00235-s001.zip › ijms-2054929-supplementary.pdf]

SUPPLEMENTARY INFORMATION

Coprinopsis cinerea galectin CGL1 induces apoptosis and inhibits tumor growth in colorectal cancer cells

Mengli Yan[1,2,3#], Yaxuan Chen[1,2,3#], Mengke Li[1,2,3], Jiamin Wu[1], Zemin Fang[1,2,3], Junjun Wang[4*] and Juanjuan Liu[1,2,3*]

1, School of Life Sciences, Anhui University;

2, Anhui Key Laboratory of Modern Biomanufacturing;

3, Anhui Provincial Engineering Technology Research Center of Microorganisms and Biocatalysis, Hefei, Anhui 230601, China

4, High Magnetic Field Laboratory, Chinese Academy of Sciences, Hefei, Anhui 230031, China

# The authors contributed equally to this work.

* Corresponding authors

Phone/Fax: +86-551-63861063

E-mail: liu_juan825@ahu.edu.cn (L. J.); Junjunwang1222@hmfl.ac.cn (W. J.).

Supplementary Methods

Dead-live staining

Cell dead-live staining assays were performed using the Calcein / PI (Propidium Iodide) cell viability assay kit (Beyotime Biotech, Shanghai, China) according to the manufacturer's instructions. Briefly, HCT116 cells were seeded into 96-well plates at 1×10^6 cells/cm^2 and allowed to adhere overnight. Then the cells were treated with 100 μg/mL CGL1 or CGL2 for 16 h, washed twice with PBS, added diluted PI and Calcein AM detection buffer, and incubated for 30 min at room temperature. After PBS washing, images were captured under an Xplore SpinSR microscope (Olympus, Japan) with excitation and emission wavelengths of 488 nm and 561 nm, respectively. All experiments were repeated at least three times and each in triplicate, and representative micrographs were shown in the Figures

Histomorphology analysis

The tissue samples of livers, kidneys, and spleens of mice were fixed in 4% neutral-buffered formalin (Servicebio, Wuhan, China). Each tissue was paraffin-embedded, sliced to a thickness of 4 μm, and then stained with hematoxylin-eosin (H&E). Images were captured with an Olympus microscope under bright field illumination and processed using Adobe Photoshop 7.0 software (Adobe Inc., San Jose, CA).

Supplementary Figures

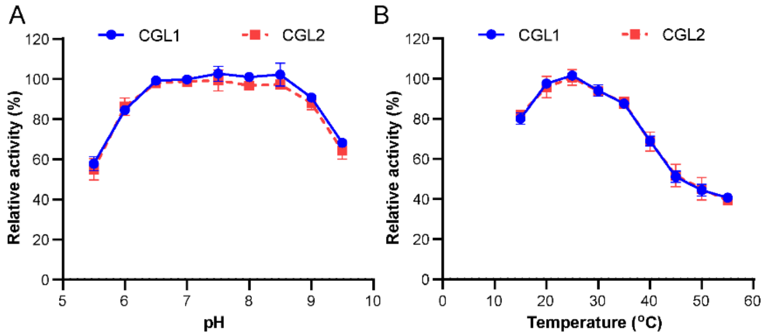

Figure S1 Effects of pHs and temperatures on the activities of CGL1 and CGL2. (A), pH optimum. Samples were incubated at 25 °C. (B), Temperature optimum. Samples were incubated at pH 7.0.

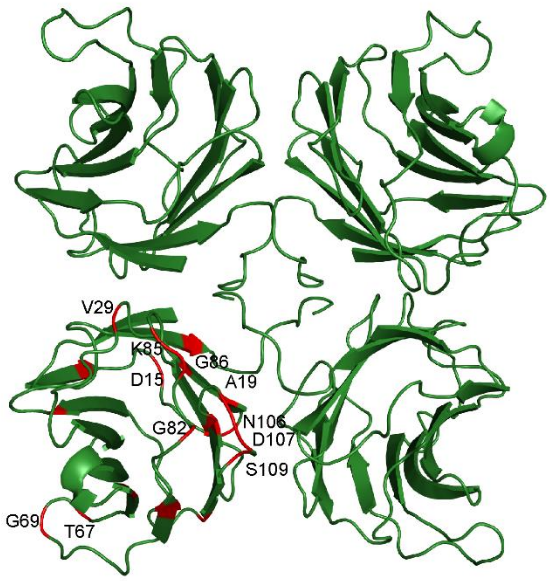

Figure S2 Computational modeling of CGL1 structure using CGL2 as the template shows CGL1 is a tetramer. The amino acids in CGL1 different from CGL2 were displayed.

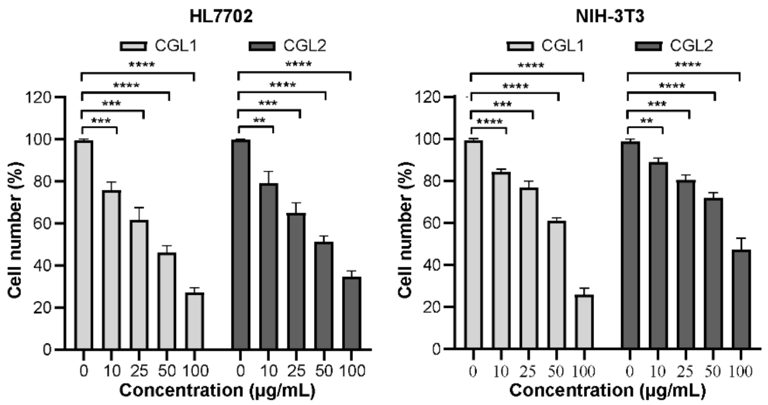

Figure S3 The cell viability of two normal cells after CGL1 and CGL2 treatment. HL7702 and NIH-3T3 cells were incubated with serials concentrations of CGL1 or CGL2 for 16 h, and then the cell viabilities were detected. The data were analyzed using a student's *t*-test (***P* < 0.01, ****P* < 0.001, *****P* < 0.0001). Data show mean ± SD, n = 3.

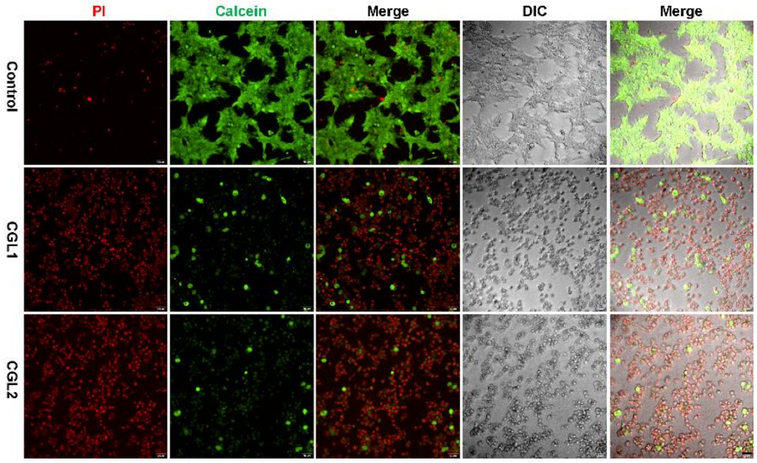

Figure S4 Dead-live staining of HCT116 cells incubated with or without 100 μg/mL CGL1 and CGL2 for 16 h. Scale bar, 50 μm.

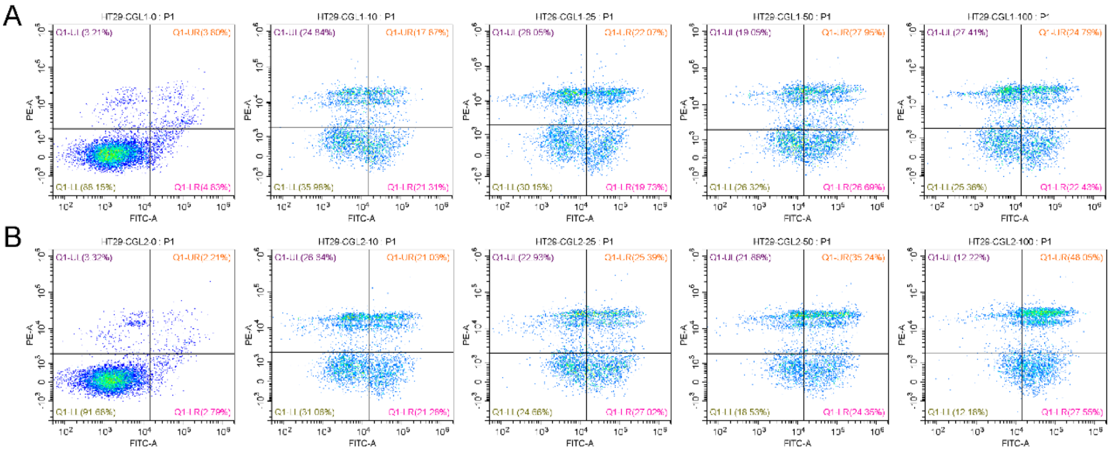

Figure S5 CGL1 and CGL2 induce HT29 cells apoptosis. HT29 cells were incubated with serials concentrations of CGL1 (A) and CGL2 (B) for 16 h, dually labeled with Annexin and PI, and then analyzed using flow cytometry.

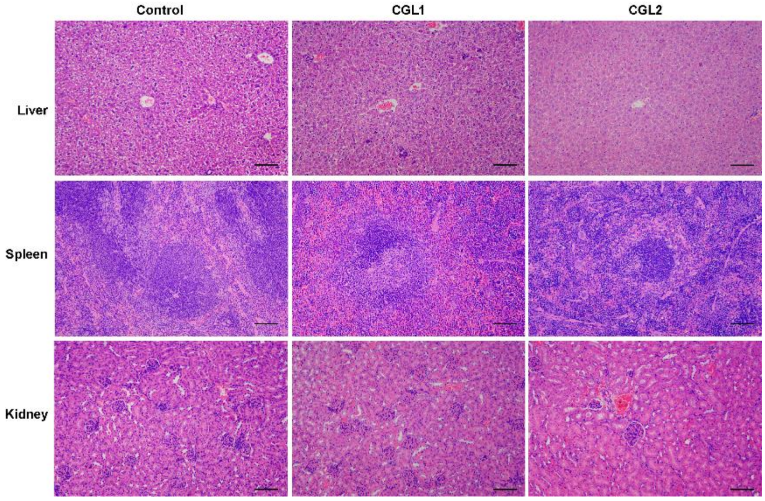

Figure S6 H&E stained livers, spleens, and kidneys of HCT116-bearing mice treated with CGL1 and CGL2 at day 14. Scale bar, 100 μm.
